# Overexpression of chromatin assembly factor-1 p60, poly(ADP-ribose) polymerase 1 and nestin predicts metastasizing behaviour of oral cancer

**DOI:** 10.1111/j.1365-2559.2012.04313.x

**Published:** 2012-12

**Authors:** Massimo Mascolo, Gennaro Ilardi, Maria Fiammetta Romano, Angela Celetti, Maria Siano, Simona Romano, Chiara Luise, Francesco Merolla, Alba Rocco, Maria Luisa Vecchione, Gaetano De Rosa, Stefania Staibano

**Affiliations:** Department of Biomorphological and Functional Sciences, Pathology SectionNaples, Italy; 1 Department of Biochemistry and Medical BiotechnologyNaples, Italy; 2 Institute of Experimental Endocrinology and Oncology ‘G. Salvatore’ EOS-CNR, CNR, c/o Department of Cellular and Molecular Biology and PathologyNaples, Italy; 3 Department of Clinical and Experimental Medicine, Gastroenterology Unit, School of Medicine, University ‘Federico II’Naples, Italy

**Keywords:** CAF-1 p60, oral cancer, PARP-1, prognosis, stem cells

## Abstract

**Aims:**

The natural history of oral squamous cell carcinomas (OSCCs) is variable and difficult to predict. This study aimed to assess the value of the expression of poly(ADP-ribose) polymerase 1 (PARP-1), chromatin assembly factor-1 (CAF-1)/p60 and the stem cell markers CD133, CD166, CD44, CD44v6 and nestin as markers of outcome and progression-free survival in OSCC patients.

**Methods:**

Clinical data were collected from 66 patients (41 male and 25 female, aged 29–92 years) who underwent surgery for OSCC of the tongue, floor, lips, and palate. During follow-up (range: 12–131 months), 14 patients experienced relapse/metastasis and/or death. The study was performed by immunohistochemistry on paraffin-embedded tumour tissues, western blot analysis of tumour protein lysates and human cell lines, and RNA silencing assays. In addition, the human papillomavirus (HPV) status of primary tumours was evaluated by immunohistochemistry and viral subtyping. Univariate and multivariate analyses were performed to determine the correlation between these parameters and the clinical and pathological variables of the study population.

**Results and conclusions:**

We found that a PARP-1^high^/CAF-1 p60^high^/nestin^high^ phenotype characterized the OSCCs with the worst prognosis (all HPV-negative). This may be of benefit in clinical management, since radio-enhancing anti-PARP-1 and/or anti-CAF-1/p60 agents may allow radioresistance to be bypassed in the nestin-overexpressing, metastasizing OSCC cells.

## Introduction

Oral squamous cell carcinoma (OSCC) is the most frequent cancer of the head and neck region. It represents the fifth most common cancer worldwide, and is the sixth most common cause of cancer-related deaths per year in the USA.^1^,^2^ The major risk factors for this tumour are prolonged exposure to tobacco and alcohol. In many cases surgery leaves patients disfigured and/or necessitates further reconstructive procedures.^1^,^3^ Unfortunately, OSCCs are frequently unresponsive to alternative therapeutic options, such as radiotherapy and chemotherapy, which target the hierarchically organized, rapidly dividing tumour cells constituting the bulk of the tumour mass.^4^,^5^ It has been hypothesized that subsets of self-renewing, proliferatively quiescent tumour cells with stem-like properties, known as cancer stem cells (CSCs), may account for the resistance to DNA-damaging agents and the failure of long-term disease control of malignant tumours.^6^–^15^ The defence strategies of cancer cells against the cytotoxic damage and apoptotic DNA double-strand breaks induced by therapeutic ionizing radiation or alkylating agents encompass the rapid synthesis and degradation of poly(ADP-ribose) by cellular poly(ADP-ribose) polymerases (PARPs).^16^–^18^ The addition of poly(ADP-ribose) groups to histones induces nucleosome modification and relaxation of the chromatin structure, facilitating DNA repair through the action of large protein complexes, such as the molecular chaperone chromatin assembly factor-1 (CAF-1).^18^–^21^ CAF-1 comprises a complex of three subunits (p150, p60, and p48) and drives the incorporation and assembly of H3K56-acetylated histones into chromatin in response to oxidative stress, DNA damage, and mismatch-containing strands, restoring chromatin structure on the completion of double-strand break repair.^22^–^27^ Knocking down p60 disrupts the activity of CAF-1 in nucleosome assembly, causing replication fork arrest, activation of the intra-S-phase checkpoint, and global defects in chromatin structure.^28^–^31^ The nuclear expression of CAF-1/p60 is increased in multiple types of cancer, proportionally to their adverse clinical behaviour.^23^,^31^–^34^ On account of these postulates, we examined the expression of the stem cell-associated antigens CD133, CD166, CD44 with the v6 variant and nestin (frequently associated with CSCs of solid malignancies) in a series of primary and metastatic OSCCs.^35^ We then compared these results with the expression levels of PARP-1 and CAF-1/p60 proteins in the same group of tumours. In addition, on the basis of evidence that a distinct cohort of head and neck cancers testing positive for high-risk (HR) human papillomavirus (HPV) DNA shows less aggressive behaviour and better response to therapy, we related our data to the HPV status of primary tumours.^36^–^39^ As HR HPVs cause strong expression of p16^INK4a^, a component of the Rb tumour suppressor gene pathway, and immunohistochemistry for p16^INK4a^ is considered to be a reasonable surrogate for detecting transcriptionally active HPV infection, p16^INK4a^ expression was also investigated in our cases of OSCC.^40^–^43^ Finally, we explored the existence of any significant association between the several parameters that we analysed and the clinical course of OSCC.

## Materials and methods

### Patients and Tissue Samples

Formalin-fixed, paraffin-embedded tissue blocks of 66 primary OSCCs (41 tongue, 16 floor, five lip and four palate squamous cell carcinomas) and corresponding metastases, diagnosed and excised with healthy surgical margins from January 2000 to December 2009, were retrieved from the archives of the Pathology Section of the Department of Biomorphological and Functional Sciences, ‘Federico II’ University of Naples. The clinical data and pathological features of the tumours are reported in Table 1.^44^ No patient experienced radiotherapy before surgery. The study design and procedures involving tissue samples collection and handling were performed according to the Declaration of Helsinki, in agreement with the current Italian law, and to the Institutional Ethical Committee guidelines.

**Table 1:** Clinical and pathological features of the study population

Stage*	Patients	Sex (male/female)	Age† (years)	Subsite	Grading	Follow-up† (months)	Clinical outcome
I	16	9/7	55.8 (30–75)	15T	2 G1	41.3 (12–66)	1D; 1R,M,D
				3L	6 G2		
				2F	8 G3		
				1P			

II	13	8/5	64.3 (29–92)	13T	9 G2	41.3 (12–92)	1M; 1D; 1R,M,D
				5F	4 G3		
				1P			

III	7	6/1	54.0 (35–75)	2T	5 G2	38.9 (13–124)	1M,D; 1R,M,D
				2F	2 G3		

IV	30	18/12	63.0 (33–89)	11T	6 G1	49.2 (12–131)	2R; 1RM; 3D; 1R,M,D
				2L	5 G2		
				7F	19 G3		
				2P			

	66	41/25	60.5 (29–92)		8 G1	44.6 (12–131)	–
				25 G2			
				33 G3			

D, Death from disease; F, floor; G, grade; L, lip; M, distant metastasis; P, palate; R, relapse; T, tongue.

* Stage classes were determined according to the American Joint Committee on Cancer (2009).^52^

† For Age and Follow-up, given numbers are respectively means and ranges.

### Tissue Microarray (TMA) Construction

The most representative tumour area for each case was selected on a donor tissue block under the guidance of the corresponding haematoxylin and eosin (H&E) section. A manual tissue microarrayer (Tissue-Tek Quick-Ray Sakura, Torrance, CA, USA) was used to punch one cylindrical core tissue specimen (3 mm diameter) from the selected area. The cores were implanted into a recipient paraffin block to construct the TMA, each containing two samples of non-neoplastic oral mucosa (control). After chilling at −10°C for 30 min,^43^ several 4-μm sections were cut from each TMA. The first section was stained with H&E to confirm both the presence of the selected areas from each tumour and the integrity of tissues. The other sections were mounted on poly-lysine coated glass slides.

### Immunohistochemistry

For each case, double immunolabelling of routine tissue sections and corresponding tissue microarrays (TMAs) was performed with the EnVision™ G/2 Doublestain System (Dako, Carpinteria, CA, USA).^45^,^46^ Immunohistochemistry was carried out as previously described.^32^–^35^,^47^ Briefly, sections were heated at 55°C for 60 min, deparaffinized, and processed for antigen retrieval by microwaving in 1% sodium citrate buffer, pH 6.0. Non-specific binding was blocked with 1.5% non-immune mouse serum (1:20; Dakopatts, Hamburg, Germany) and endogenous peroxidase and alkaline phosphatase activities were quenched with dual endogenous enzyme block (0.5% H_2_O_2_ in methanol and detergent). Sections were then incubated with primary antibodies (Table 2) followed by the appropriate secondary antibody, and the reaction was detected using 3,3′-diaminobenzidine (Vector Laboratories, Burlingame, CA, USA) and permanent red; nuclei were counterstained with Mayer’s haematoxylin. For each run, positive and negative controls were included (Table 2).^33^–^35^,^47^,^48^ For negative controls, non-immune serum in T-TBS buffer (1:500) was used instead of the primary antibodies. A brown signal confined to the nucleus indicated immunopositivity for either CAF-1/p60 or PARP-1. Positivity for nestin, CD44, CD44v6, CD133 and CD166 was visualized as red membrane and/or cytoplasmic staining. For all primary antibodies, the level of immunostaining was scored semiquantitatively; for each marker, grouping into low and high expression was established on the basis of median values (Table 2).^31^–^34^,^48^ Immunohistochemical screening for HPV status was performed using the p16^INK4a^-CINtec histology kit (E6H4; mtm Laboratories, Heidelberg, Germany).^49^,^50^

**Table 2:** Primary antibodies used for immunohistochemistry

Antibody*	Clone number	Manufacturer	Dilution	Positive control	Staining pattern	Median %	Score
CAF-1/p60	SS53	Abcam, Cambridge, MA, USA	1:300	Prostate carcinoma	N	17.5	Low: <17.5%
							High: ≥17.5%

CD133/1	AC133	Miltenyi Biotec, Auburn, CA, USA	1:100	Hair follicle	M	9.5	Low: <9.5%
							High: ≥9.5%

PARP-1	F-2	Santa Cruz Biotechnology, Santa Cruz, CA, USA	1:200	Human lymphoma	N	15	Low: <15%
							High: ≥15%

Nestin	10c2	Santa Cruz Biotechnology, Santa Cruz, CA, USA	1:100	Human brain tissue and normal skin	C	6	Low: <6%
							High: ≥6%

CD44 (H-CAM)	DF1485	Novocastra/Leica, Newcastle upon Tyne, UK	1:50	Human tonsil	M	16	Low: <16%
							High: ≥16%

CD44v6	VFF-7	Novocastra Laboratories, Newcastle upon Tyne, UK	1:50	Human tonsil	M	10%	Low: <10%

CD166 (ALCAM)	MOG/07	Vector Laboratories, Burlingame, CA, USA	1:100	Normal skin	M/C	9.5	Low: <9.5%
							High: ≥10%
							High: ≥9.5%

p16^INK4a^	E6H4	mtm Lab. AG, Heidelberg, Germany	Ready-to-use	CIN3	N/C		Binary rating system, ‘positive’ and ‘negative’†

C, Cytoplasmatic signal; M, membrane signal; N, nuclear signal.

* All antibodies used were mouse monoclonal.

† Positive: diffuse staining (>50% of neoplastic cells).

### HPV Genotyping

DNA isolation and HPV genotyping were performed using the INNO-LiPA polymerase chain reaction (PCR)-based HPV Genotyping Extra test (Innogenetics Biotechnology for Healthcare, Gent, Belgium).^51^,^52^ This test identifies 28 HPV genotypes: 6, 11, 16, 18, 26, 31, 33, 35, 39, 40, 43, 44, 45, 52, 53, 54, 56, 58, 59, 66, 68, 69, 70, 71, 73, 74, and 82. Viral subtyping was performed only in p16^INK4a^-positive cases.

### Cell Culture

Normal human keratinocytes (HNEK) were cultured in keratinocyte growth medium (Cambrex, East Rutherford, NJ, USA). BHY, CAL33 and HN cell lines are described elsewhere.^53^ HN and BHY cell lines were derived from a human oral cavity SCC, and CAL33 cells from human tongue SCC. Cells were maintained in DMEM supplemented with 10% fetal bovine serum, 2 mm l-glutamine and 100 units/ml penicillin-streptomycin (GIBCO, Paisley, PA, USA). The HaCat cells were derived from in vitro spontaneously transformed keratinocytes from histologically normal skin.

### Western Blotting

Protein expression analysis on HN, BHY and CAL33 cell lines was performed according to standard procedures.^53^ For tissue protein extraction, samples were snap-frozen and immediately homogenized in lysis buffer using the Mixer Mill apparatus (Qiagen, Germantown, Philadelphia, PA, USA). Protein was quantified using a modified Bradford assay (Bio-Rad, Hercules, CA, USA). Antigens were revealed using an enhanced chemiluminescence detection kit (ECL; Amersham, Little Chalfont, UK).

### Cell Death Assay

HaCat and CAL 33 cells were transfected with specific short interfering oligoribonucleotide (siRNA), corresponding to human cDNA sequences for CAF-1 p60 (Qiagen) or with a non-silencing RNA (AllStars negative control siRNA; Qiagen) as control. For transfection details see ref. 54. After 24 h, cells were treated with the PARP-1 inhibitor PJ34 (Alexis, Vinci-Biochem, Firenze, Italy) at doses of 0.5, 5 and 50 μm. Analysis of DNA content was performed by propidium iodide incorporation as described.^54^

### Statistical Analysis

The selected predictor variables were compared by use of the *χ*^2^ test or Fisher’s test, and correlations between data were analysed with the Spearman rank correlation test. For comparison of the event-free survival time (events: relapse, metastasis, or death) between two categories of individuals, the log-rank Mantel–Haenszel test was applied. A two-tailed test of significance with a *P*-value <0.05 was considered to be statistically significant. Multivariate analyses were performed to correlate different markers; the Bonferroni corrected *P*-value was considered to be significant if it was <0.05. The statistical analysis was performed using the r statistical package v. 2.10.1 (R Foundation for Statistical Computing, Vienna, Austria). Evaluation of the intraobserver and interobserver agreement for the tested proteins on whole sections and TMAs and of the concordance between sections and TMAs was performed by use of Cohen’s weighted kappa statistic.^55^

## Results

### Immunohistochemical Staining of TMAs and Routine Tissue Sections

All of the antibodies used in this study showed a high concordance level between the expression values on routine sections and those on TMA sections, as reported below. For each antibody, we report the results of the immunohistochemical staining, without specifying whether the data refer to TMA sections or whole sections of tumours. The immunohistochemistry results for each antibody analysed are reported in detail in Table 3.

**Table 3:** Immunohistochemical staining and HPV genotyping results, tumour staging and follow-up of patients

	I	II	III	IVA	Follow-up
CAF-1/p60					
Low	15	5	3	10	33W

High	6	14	1	12	19W; 2R; 1M; 5D; 1R,M; 1M,D; 4R,M,D

CD133					
Low	13	6	3	11	31W; 2R,M,D

High	8	13	1	11	21W; 2R; 1M; 5D; 1R,M; 1M,D; 2R,M,D

PARP-1					
Low	9	8	2	9	28W

High	12	11	2	13	24W; 2R; 1M; 5D; 1R,M; 1M,D; 4R,M,D

Nestin					
Low	11	6	3	9	29W

High	10	13	1	13	23W; 2R; 1M; 5D; 1R,M; 1M,D; 4R,M,D

CD44					
Low	12	6	2	10	28W; 2R

High	9	13	2	12	24W; 1M; 5D; 1R,M; 1M,D; 4R,M,D

CD44v6					
Low	7	3	1	4	13W; 2R

High	14	16	3	18	39W; 1M; 5D; 1R,M; 1M,D; 4R,M,D

CD166/ALCAM					
Low	14	7	3	10	33W; 1D

High	7	12	1	12	19W; 2R; 1M; 4D; 1R,M; 1M,D; 4R,M,D

p16^INK4a^					
Negative	14	18	2	21	42W; 2R; 1M; 5D; 1R,M; 1M,D; 4R,M,D

Positive	6*	1*	2*	1*	10W

D, Death from disease; M, metastasis; R, relapse; W, alive and well.

* All the p16^INK4a^-positive tumours were positive for HPV16, as assessed by the INNO-LiPA polymerase chain reaction (PCR)-based HPV Genotyping Extra test.

**Figure 1:**
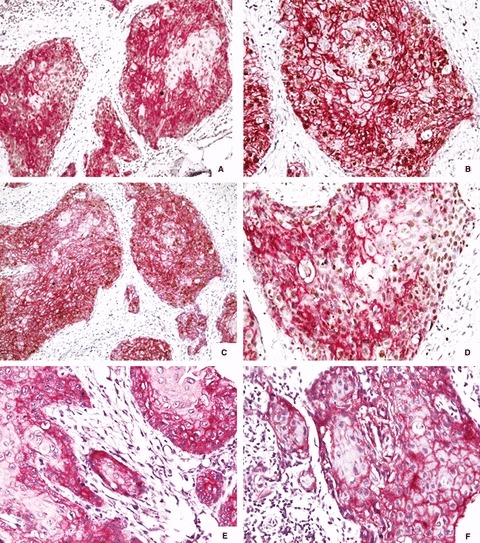
A case of metastasizing oral squamous cell carcinoma. **A**,**B**, Strong immunostaining for PARP-1 (nuclear) and CD166 (cytoplasmic and cell membrane). **C**,**D**, Diffuse immunostaining for CAF-1/p60 (nuclear) and CD44 (cytoplasmic and cell membrane). **E**,**F**, Extensive, strong positivity for nestin (cytoplasmic and cell membrane).

**Figure 2:**
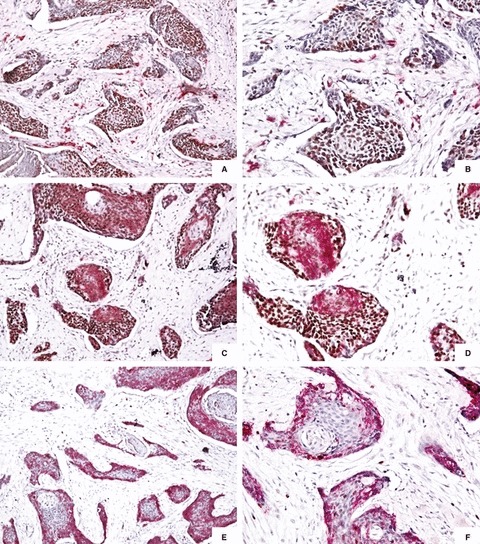
A case of oral squamous cell carcinoma, N0 M0, at the end of follow-up. **A**,**B**, Low to moderate immunohistochemical expression of nestin and CAF-1 p60. **C**,**D**, Weak immunopositivity for CD44 and CAF-1/p60. **E**,**F**, Weak staining for CD166 and PARP-1.

CAF-1/p60, PARP-1 and nestin were expressed in all OSCCs; tumours were assigned to two (low-score and high-score) categories on the basis of the median values of marker expression (Table 2). All the tumours showing the simultaneous overexpression of CAF-1/p60, PARP-1 and nestin, developed, during follow-up, recurrence and/or metastases or death (Figure 1; Table 3). This overexpression was retained by the corresponding metastatic tissues (Figure 1). Among the 36 cases showing high-level CD44 expression, only 12 (33%) developed an adverse event during follow-up (Table 3). The immunohistochemical expression of CD166, CD133 and CD44v6 was variable, and did not show a significant association with clinical behaviour. The p16^INK4a^-positive tumours were all positive for HPV16 (Table 3). Among p16^INK4a^-positive tumours, CAF-1/p60, PARP-1 and nestin were only barely detectable (Figure 2). In TMAs, as in routine sections, focal immunoreactivity for the evaluated proteins was observed in a few keratinocytes of normal oral epithelium, almost always localized to the basal layer.

### Protein Expression of Stem Cell Markers, PARP-1 and CAF-1/p60 IN OSCC and Cell Lines

**Figure 3:**
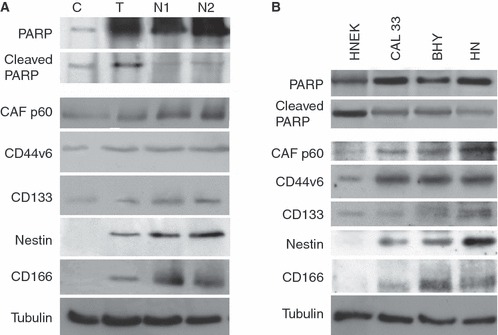
Expression of stem cell markers, PARP-1 and CAF-1/p60 in oral squamous cell carcinoma (OSCC) and cell lines. **A**, Snap-frozen OSCC, protein lysates: high expression level of uncleaved p111 PARP-1 and CAF-1/p60 in primary OSCC (T) and corresponding metastases (N1, N2). All of the evaluated stem cell markers were found to be expressed in OSCC. However, the highest levels were registered for nestin, particularly in metastases. Tubulin detection was used to confirm equal gel loading. **B**, Cultured human OSCC lines: OSCC cell lines overexpressed CAF-1/p60 and PARP-1. Among stem cell markers, nestin, CD44, CD44v6 and CD166 showed the highest expression levels. A primary culture of HNEK cells was used as control.

To verify the immunohistochemical data, we harvested protein lysates from selected high-stage/grade snap-frozen OSCC samples (*n* = 10) and from the corresponding normal mucosa, and examined CAF-1/p60 protein levels by immunoblotting. We found CAF-1/p60 protein to be highly expressed in all carcinomas, particularly in metastatic tissue (Figure 3). The uncleaved p111 PARP-1 isoform was present in large amounts in tumours and metastases, as compared with normal counterparts (Figure 3). We previously analysed the same group of OSCC primary samples, and found high levels of CD44 and its v6 variant, which was particularly expressed in high-grade tumours. In the present study, these samples were also evaluated for the expression of nestin, CD133, and CD166. These stem cell markers showed greater expression in OSCC than in normal oral mucosa; the highest levels were registered for nestin, particularly in OSCC metastases (Figure 3). We also evaluated the expression levels of CAF-1/p60, PARP-1 and stem cell markers in some cultured human OSCC lines. CAF-1/p60 and PARP-1 were expressed at the highest level in OSCC cell lines (Figure 3), which also showed high levels of CD44v6, nestin and CD166 expression (Figure 3).

### Fluorescence-Activated Cell Sorting Analysis of OSCC Cells Upon Treatment with CAF-1/p60 siRNA and PARP-1 Inhibitor PJ34

**Figure 4:**
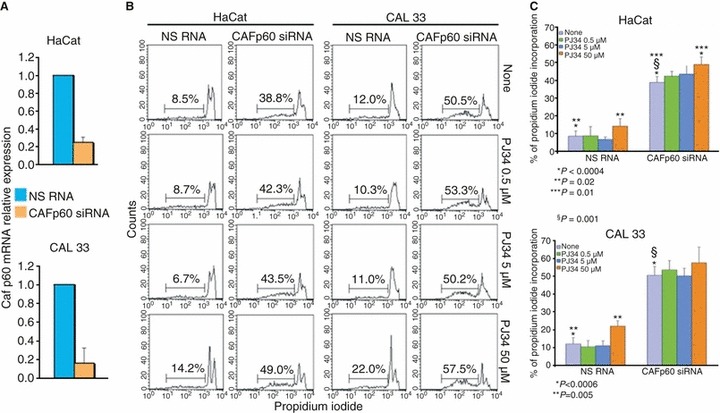
CAF-1 p60 silencing activates keratinocyte cell death: flow cytometric histograms of propidium iodide incorporation of HaCat and CAL33 cells, transfected with non-silencing (NS) RNA or CAF-1 p60 small interfering RNA. Twenty-four hours after transfection, cells were treated with the PARP-1 inhibitor PJ34, and were harvested after a further 24 h. Bars indicate hypodiploid cell percentages (**B**). Graphic representations are given of the mean values and standard deviations of cell death values (*n* = 6) for HaCat and CAL33 cells (**C**). The CAF-1/p60 siRNA efficiency was assessed by measuring the levels of CAF-1/p60 mRNA, by Real time PCR (**A**).

PJ34 at 50 μm induced cell death in both HaCat and CAL33 cells (Figure 4: HaCat, *P* = 0.02; CAL33, *P* = 0.005). CAF-1/p60 siRNA induced cell death in both cell lines (HaCat, *P* < 0.0004; CAL33, *P* < 0.0006). The extent of CAF-1/p60 siRNA-induced apoptosis was higher in CAL33 cells than in HaCat cells (*P* < 0.001). A cooperative effect between PJ34 and CAF-1/p60 siRNA was observed in HaCat cells but not in CAL33 cells. In fact, addition of 50 μm PJ34 to CAF-1/p60-silenced CAL33 cells resulted in a significant increase in cell death (Figure 4: *P* = 0.01).

### Statistical Analysis

The level of agreement for the immunohistochemical staining evaluation, expressed by the kappa coefficient, was >0.75 for both intraobserver and interobserver evaluations, on sections and on TMAs, for all of the antibodies used in the present study. The concordance between the expression levels evaluated on the whole sections and on TMA sections was high (kappa coefficient >0.75).

No statistical differences for age, gender, tumour subsite, grade or stage of disease were found between patients who had different clinical outcomes (Table S1). CAF-1/p60, PARP-1, CD166, nestin, CD44 and CD44v6 were expressed at significantly higher levels in patients who had an adverse event during the follow-up. CAF-1/p60, PARP-1 and nestin showed the highest sensitivity (1.00) and specificity (0.64, 0.54, and 0.56, respectively) (Table 4). Similar results were obtained on evaluating these multiple markers against a single adverse event (Tables S2–S4). As expected, the various markers that we investigated were statistically correlated with each other (Tables 5 and 6). We focused on the three proteins (CAF-1/p60, PARP-1, and nestin) that showed the strongest correlation with each other (Table 5) and with tumour biological behaviour. They were significantly associated with adverse events (*P* < 0.001), showing sensitivity or specificity levels that reached 1.00. The 14 patients who had simultaneous maximum expression of CAF-1/p60, PARP-1 and nestin had at least one adverse event, with an overall median event-free time of 12 months. In contrast, the 52 patients with low expression of at least one of the three proteins in their primary tumours had a favourable outcome (Table 7). Nineteen patients showed high CAF-1/p60 and low PARP-1 and/or nestin expression; none of them had any adverse event during the follow-up. Table 8 summarizes our findings: the simultaneous ‘triple-high expression’ of CAF1 /p60, PARP-1 and nestin correlates, with high sensitivity and specificity, with relapse, metastasis, and death.

**Table 4:** Correlation between marker expression and the occurrence of an adverse event

		No event, no. (%)	Any event, no. (%)	Difference	Sensitivity	Specificity
CAF-1/p60	Low	33 (63)	0 (0)	*P* < 0.0001	1.00	0.64
				
	High	19 (37)	14 (100)			

PARP-1	Low	28 (54)	0 (0)	*P* < 0.0001	1.00	0.54
				
	High	24 (46)	14 (100)			

p16	0	42 (81)	14 (100)	NS	0.00	0.81
				
	1	10 (19)	0 (0)			

HPV	0	42 (81)	14 (100)	NS	0.00	0.81
				
	1	10 (19)	0 (0)			

CD166	Low	33 (63)	1 (7)	*P* < 0.0001	0.93	0.64
				
	High	19 (37)	13 (93)			

CD133	Low	31 (60)	2 (14)	*P* = 0.0001	0.86	0.60
				
	High	21 (40)	12 (86)			

Nestin	Low	29 (56)	0 (0)	*P* < 0.0001	1.00	0.56
				
	High	23 (44)	14 (100)			

CD44	Low	28 (54)	2 (14)	*P* < 0.0001	0.86	0.54
				
	High	24 (46)	12 (86)			

CD44v6	Low	13 (25)	2 (14)	*P* < 0.0001	0.86	0.25
				
	High	39 (75)	12 (86)			

NS, Not significant.

**Table 5:** Correlations between expression of markers

	CAF-1/p60	PARP-1	p16	HPV	CD166	CD133	Nestin	CD44
PARP-1	0.71***	1						

p16	−0.09	−0.27	1					

HPV	−0.09	−0.27	1***	1				

CD166	0.61***	0.65***	−0.36*	−0.36*	1			

CD133	0.02	0.24	−0.13	−0.13	0.21	1		

Nestin	0.61***	0.79***	−0.27*	−0.27*	0.63***	0.16	1	

CD44	0.07	0.18	−0.35*	−0.35*	0.45***	0.23	0.30*	1

CD44v6	0.21***	0.35*	−0.32*	−0.32*	0.41*	0.24*	0.39*	0.65***

* *P* < 0.05;

*** *P* < 0.001.

**Table 6:** Pairwise comparisons between CAF-1 p60, PARP-1, nestin, CD166, CD133, CD44 and CD44v6 expression

		Mean difference	Standard error	P*	95% CI
CAF-1/p60 versus	PARP1	−6.197	1.578	0.0044	−11.188 to −1.206
	
	CD166	8.515	1.050	<0.0001	5.194 to 11.836
	
	CD133	9.212	1.061	<0.0001	5.857 to 12.567
	
	Nestin	9.030	1.136	<0.0001	5.440 to 12.621
	
	CD44	3.955	1.348	0.0973	−0.309 to 8.218
	
	CD44v6	8.318	1.307	<0.0001	4.187 to 12.450

PARP-1 versus	CD166	14.712	1.834	<0.0001	8.914 to 20.510
	
	CD133	15.409	2.067	<0.0001	8.874 to 21.945
	
	Nestin	15.227	1.647	<0.0001	10.018 to 20.436
	
	CD44	10.152	2.388	0.0015	2.600 to 17.703
	
	CD44v6	14.515	2.355	<0.0001	7.069 to 21.961

CD166 versus	CD133	0.697	0.863	1.0000	−2.031 to 3.425
	
	Nestin	0.515	1.153	1.0000	−3.130 to 4.161
	
	CD44	−4.561	1.110	0.0024	−8.070 to −1.051
	
	CD44v6	−0.197	1.171	1.0000	−3.900 to 3.506

CD133 versus	Nestin	−0.182	1.341	1.0000	−4.421 to 4.057
	
	CD44	−5.258	0.773	<0.0001	−7.702 to −2.813
	
	CD44v6	−0.894	0.771	1.0000	−3.333 to 1.545

Nestin versus	CD44	−5.076	1.550	0.0356	−9.976 to −0.175

	CD44v6	−0.712	1.533	1.0000	−5.559 to 4.135

CD44 versus	CD44v6	4.364	0.438	<0.0001	2.978 to 5.749

CI, Confidence interval.

* Bonferroni corrected.

**Table 7:** Expression levels for CAF-1/p60, PARP-1 and nestin

CAF-1	PARP-1	Nestin	*N*	R	M	D	RM	MD	RMD
Low	Low	Low	7	0	0	0	0	0	0
		
		High	9	0	0	0	0	0	0
	
	High	Low	10	0	0	0	0	0	0
		
		High	7	0	0	0	0	0	0

High	Low	Low	5	0	0	0	0	0	0
		
		High	7	0	0	0	0	0	0
	
	High	Low	7	0	0	0	0	0	0
		
		High	14	2	1	5	1	1	4

D, Death from disease; M, metastasis; *N*, number count; R, relapse.

**Table 8:** Correlation between CAF-1 p60, PARP-1 and nestin ‘triple-high’ expression and the occurrence of an adverse event

	Any event	R	M	D
*P*	0.00	<0.0001	<0.0001	<0.0001

Sensitivity	1.00	1.00	1.00	1.00

Specificity	1.00	0.88	0.88	0.93

D, Death from disease; M, metastasis; R, relapse.

## Discussion

Our study provides evidence for the existence of a definite inter-relationship between the overexpression of PARP-1, CAF-1/p60 and nestin and the biological aggressiveness of OSCCs. Such a finding seems to prove that these molecules may serve as novel predictive biomarkers for chemotherapy/radiotherapy responsiveness and for the prognosis of these lethal cancers, shedding new light on the very complex molecular events underlying the neoplastic progression of OSCCs.

### Tumour Heterogeneity and Expression of Stem Cell Markers in OSCCs

The presence of non-cycling CSCs, which are inherently resistant to chemotherapy and radiotherapy, greatly contributes to the metastasizing ability of cancers.^13^–^15^,^56^–^58^ Unfortunately, CSCs may be heterogeneous from patient to patient and within each tumour type and single tumour clones.^56^,^59^–^62^ Moreover, given that, currently, specific markers are not able to unequivocally distinguish CSCs from their differentiating progeny and their normal tissue counterparts, the goal of specifically targeting CSCs, avoiding unwanted toxicity to normal stem cells, seems far from being reached.^13^,^63^ The metastasizing OSCCs of our study showed high cellular levels of CD133, CD44, CD44v6, and CD166; their coexpression, rather than being specifically clustered in a constant fashion, was randomly distributed. This finding is reminiscent of the variable combination of expression between a stem cell marker (CD44) and other stem cell markers, as recently reported for head and neck squamous cell carcinoma, and fits with the hypothesis that CSC evolution is a continuous dynamic process, which constantly leads to new generations of CSCs with genetic and/or epigenetic changes favouring metastatic potential.^13^,^64^ Among all of the stem cell markers that we evaluated, only nestin was maximally expressed in 100% of metastasizing OSCCs. According to this point of view, the nestin^high^ CSCs should be considered to be the result of the selection of a metastatic clone among the heterogeneous pool of OSCC cells expressing stem cell markers.

### PARP-1 and CAF-1/p60 Expression in OSCCs

PARP-1 and CAF-1/p60 nuclear proteins cross-talk with several molecular pathways involved in histone acetylation. The poly(ADP-ribosyl)ation of histones effectively functions like acetylation, maintaining chromatin nucleosomes in a fully relaxed, transcriptionally active state. Either PARP-1^65^–^68^ or CAF-1/p60^31^–^34^ overexpression has been reported in multiple types of cancer, and this has been related to histopathological grade and/or adverse clinical behaviour. Their expression could help tumour cells to withstand genotoxic stress, by increasing their resistance to DNA-damaging agents, and may result in radoresistance and chemoresistance. In the OSCCs analysed, the metastasizing group showed striking immunoreactivity for both CAF-1/p60 and PARP-1, either on tissue sections or in tumour protein lysates. Accordingly, the protein expression analysis of HN, BHY and CAL33 cells showed increased amounts of CAF-1/p60 and PARP-1 in OSCCs with respect to controls. These data are in agreement with previous reports of CAF-1/p60 and PARP-1 as markers of adverse biological behaviour in oral and head and neck squamous cell carcinomas.^35^,^69^ Moreover, we found that full-length PARP-1 increased progressively from controls to primary tumours and cancer cell lines, constituting the only detectable PARP-1 form in metastatic tissue. This finding is in line with results from previous studies performed by our team on human skin melanomas, showing the existence of a direct correlation between melanoma cell aggressiveness and overexpression of full-length PARP-1, evaluated by western blot analysis, and with the reported strong reduction of PARP-1 cleavage in cisplatin-resistant melanoma cell lines.^70^ As in the case of skin melanoma, our findings suggest that inhibition of the PARP-1-mediated apoptotic process may also be involved in the worst behaviour of a subset of OSCCs, as the cleaved form of PARP-1 is nearly undetectable in metastatic cases. As is common knowledge, cleavage of the stable 85-kDa fragment from full-length PARP-1 is a caspase-mediated early apoptotic event. Conversely, full-length PARP-1 facilitates the survival of proliferating cells under conditions of DNA damage, mainly via DNA base-excision repair, acting as a negative regulator of genome instability in both normal and neoplastic cells.^71^–^73^ Bürkle *et al.*^74^ showed that overexpression of full-length, wild-type PARP-1 in transfected hamster cells led to a striking cytoprotective effect, by suppression of DNA damage-induced genomic instability in proliferating cells exposed to genotoxic stress. Moreover, a recent meta-analysis carried out in a large retrospective gene expression dataset revealed that both PARP-1 mRNA and PARP-1 evaluated by immunohistochemistry were overexpressed in the subset of breast cancer with the worst prognosis, in terms of metastasis-free survival and overall survival. This led to the conclusion that nuclear PARP-1 overexpression is an independent prognostic factor for disease-free and overall survival of patients. They hypothesized that PARP-1 overexpression, in some cases, may result from defective PARP-1 cleavage, resulting in reduced tumour apoptosis (see also the previous report of Tang *et al.*).^72^,^75^ Similar results were obtained in a study of ovarian cancer.^76^ We have demonstrated, for the first time, that metastasizing OSCCs are characterized by constant high expression of PARP-1 (almost entirely full-length) coupled to hyperexpression of CAF-1/p60 and nestin, regardless of the grade and size of tumours at diagnosis.

### HPV Status and Molecular Phenotype of OSCCs

The link between persistent HR HPV infection and the development of preneoplastic and cancerous lesions of human mucosal epithelia is well known. In head and neck cancers, a clear relationship between HR HPV infection and oropharyngeal cancer has been shown, whereas the data concerning the link between HPV and oral cancer are still not conclusive. The reported prevalence of HPV in oral epithelial neoplastic and preneoplastic lesions ranges from 30% to 86%.^77^–^82^ This could be attributable to the lack of standardization of the criteria used for population selection among the different studies. In addition, a meta-analysis of 94 studies investigating 4680 OSCCs revealed that a significantly higher frequency of HPV infection was found if PCR techniques were used than when Southern blot, dot blotting and *in-situ* hybridization were employed.^77^ Nevertheless, these findings are in agreement with the hypothesis that HPV may act as an initiator of epithelial proliferation in oral carcinogenesis, independent of the anatomical subsite (tongue, gingiva, cheek, and oral floor).^77^,^78^,^81^,^83^ Our data provide useful information about the molecular events underlying the biology of HPV-positive OSCC.^84^ The 10 HPV-positive OSCCs of our series had low to moderate expression of CAF-1/p60 and PARP-1, and never showed statistically significant immunoreactivity for stem cell markers. This observation may contribute to explaining the high rate of responsiveness to radiotherapy of HPV-positive head and neck and oral squamous cancers. HR HPV E7 oncoprotein binds and degrades Rb protein, leading to an increase in p16^INK4a^ levels and to deregulated tumour cell proliferation.^43^,^85^ Stem cells normally reside in a hypoxic niche, where self-renewal and differentiation activity are balanced. When cell proliferation becomes a dominant feature, the expansion of progenitor cells can also occur. This could result in stem cell pool exhaustion.^86^ The barely detectable level of HPV-positive OSCC cells with a CSC phenotype may then represent the consequence of continuous HPV-induced p16^INK4a^ overexpression which might, in the long term, cause defects in the maintenance of stem cell self-renewal ability in these tumours. This hypothesis is in line with the reported long time lag (from 15 to 30 years) between an oral HPV infection and the development of HPV-related OSCC, and with the reported major impact of p16^INK4a^ expression on response to treatment and overall survival of patients with head and neck cancer treated using conventional radiotherapy.^87^,^88^ Low levels of PARP-1 and CAF-1/p60 with the absence of a significant number of HPV-positive tumour cells showing a CSC phenotype could therefore explain the reduced DNA-repair ability and radioresistance that characterizes this subgroup of OSCCs.

### Prognostic and Therapeutic Implications

The significant association between the PARP-1^high^/CAF-1 p60^high^/nestin^high^ phenotypes allows us to hypothesize that it could represent an epiphenomenon of the metastasizing CSC (mCSC) compartment of HPV-negative OSCCs. This finding may have relevance for clinical practice. In fact, it is now recognised that inhibition of PARPs leads to impairment of DNA double-strand-break repair, enhancing the cytotoxic effects of ionizing radiation and DNA-damaging chemotherapeutic agents.^16^,^89^,^90^ In addition, CAF-1/p60 has recently emerged as a promising target, inhibition of which could lead to cell death in aggressive tumours.^32^–^36^ This hypothesis has been further supported by the results of the cell death assay on HaCat and CAL33 cells that we performed with CAF-1 p60 siRNA and the PARP-1 inhibitor PJ34. Both PJ34 and CAF-1/p60 siRNA exerted a striking effect in each cell line, as compared with untreated cells, that was most marked in CAL33 cells. This result suggests that the cooperative effect between PARP-1 and CAF-1/p60 inhibitors is dampened in malignancy. In the near future, the association of ionizing radiation with radio-enhancing anti-PARP-1 and/or anti-CAF-1/p60 agents may provide an opportunity to bypass the radioresistance of OSCC mCSCs, minimizing side effects in surrounding normal tissues.^69^,^91^ OSCC immunohistochemical screening for PARP-1^high^/CAF-1 p60^high^/nestin^high^ tumours could identify the highly radioresistant/chemoresistant cancers that would benefit from new molecular therapies, allowing a reduction in the severe morbidity and poor long-term survival of OSCC patients.^92^ It is clear that there is still much to learn about OSCC biology. The occurrence of subsets of phenotypically distinct CSCs in primary tumours has been correlated with their aggressive behaviour.^13^ However, the ultimate role of heterogeneity within the cancer cell population in determining tumour biology and the response to radiotherapy and chemotherapy remains to be fully clarified, before definitive decisions about the best stratification factors for personalized algorithms of OSCC treatment can be made.^93^ Improved refining of the CSC population phenotype is necessary. We are aware that the arbitrariness of the cut-off scores for determining the positivity of potential tumour markers can hamper their prognostic value. So far, standardized scoring systems for evaluating immunohistochemistry in OSCC are lacking. It has been demonstrated that evaluation of immunoreactivity by using the percentage of positive tumour cells may be considered a reproducible scoring method with strong interobserver agreement. We preferred, instead, to use an unbiased method such as the median, to avoid potential bias through the use of less appropriate thresholds. Observer variation reached the acceptable standard for accurate assessment of protein expression.^94^

To the best of our knowledge, this is the first report of simultaneous overexpression of CAF-1 p60, PARP-1 and stem cell markers in a malignant tumour. Our data indicate that the interaction between PARP-1, CAF-1/p60 and nestin may constitute a specific hallmark of the aggressiveness of OSCCs. The high concordance between the expression levels of all of the evaluated proteins in whole sections and in TMAs may allow the rapid extension of the immunohistochemical evaluation of these markers to larger series of cases, favouring the adoption of screening for these proteins in the clinical setting. This may have important consequences for OSCC patients, providing us with novel candidate drug targets for reducing the number of deaths caused by the ineffective treatment of metastatic disease.

## Abbreviations

CAF-1: chromatin assembly factor-1
CSC: cancer stem cell
HPV: human papillomavirus
HR: high-risk
mCSC: metastasizing cancer stem cell
OSCC: oral squamous cell carcinoma
PARP: poly(ADP-ribose) polymerase
PCR: polymerase chain reaction
siRNA: small interfering RNA
TMA: tissue microarray

## Supporting Information

**Table S1.** Correlation between sex, age, localization, grading and staging and an adverse event (recurrence, metastasis, death from disease).

**Table S2.** Correlation between expression of all markers and relapse.

**Table S3.** Correlation between expression of all markers and metastasis.

**Table S4.** Correlation between expression of all markers and death from disease.

## References

[1] Deshpande AM, Wong DT (2008). Molecular mechanisms of head and neck cancer. Expert Rev. Anticancer Ther.

[2] Jemal A, Siegel R, Xu J (2010). Cancer statistics, 2010. CA Cancer J. Clin.

[3] Mydlarz WK, Hennessey PT, Califano JA (2010). Advances and perspectives in the molecular diagnosis of head and neck cancer. Expert Opin. Med. Diagn.

[4] Schuller DE, Wilson HE, Smith RE, Batley F, James AD (1983). Preoperative reductive chemotherapy for locally advanced carcinoma of the oral cavity, oropharynx, and hypopharynx. Cancer.

[5] Al-Sarraf M (1983). Chemotherapy strategies in squamous cell carcinoma of the head and neck. Crit. Rev. Oncol. Hematol.

[6] Monroe MM, Anderson EC, Clayburgh DR, Wong MH (2011). Cancer stem cells in head and neck squamous cell carcinoma. J. Oncol.

[7] Bao S, Wu Q, McLendon RE (2006). Glioma stem cells promote radioresistance by preferential activation of the DNA damage response. Nature.

[8] McCord AM, Jamal M, Williams ES, Camphausen K, Tofilon PJ (2009). CD133+ glioblastoma stem-like cells are radiosensitive with a defective DNA damage response compared with established cell lines. Clin. Cancer Res.

[9] Hong SP, Wen J, Bang S, Park S, Song SY (2009). CD44-positive cells are responsible for gemcitabine resistance in pancreatic cancer cells. Int. J. Cancer.

[10] Diehn M, Clarke MF (2006). Cancer stem cells and radiotherapy: new insights into tumor radioresistance. J. Natl Cancer Inst.

[11] Bertolini G, Roz L, Perego P (2009). Highly tumorigenic lung cancer CD133+ cells display stem-like features and are spared by cisplatin treatment. Proc. Natl. Acad. Sci. USA.

[12] Al-Assar O, Muschel RJ, Mantoni TS, McKenna WG, Brunner TB (2009). Radiation response of cancer stem-like cells from established human cell lines after sorting for surface markers. Int. J. Radiat. Oncol. Biol. Phys.

[13] Alison MR, Lim SML, Nicholson LJ (2011). Cancer stem cells: problems for therapy?. J. Pathol.

[14] Oliveira LR, Oliveira-Costa JP, Araujo IM (2010). Cancer stem cell immunophenotypes in OSCC. J. Oral Pathol. Med.

[15] Zhou ZT, Jiang WW (2008). Cancer stem cell model in OSCC. Curr. Stem Cell Res. Ther.

[16] Plummer R, Jones C, Middleton M (2008). Phase I study of the poly(ADP-ribose) inhibitor, AG014699, in combination with temozolomide in patients with advanced solid tumors. Clin. Cancer Res.

[17] Plummer ER (2006). Inhibition of poly(ADP-ribose) polymerase in cancer. Curr. Opin. Pharmacol.

[18] D’Amours D, Desnoyers S, D’Silva I, Poirier GG (1999). Poly(ADP-ribosyl)ation reactions in the regulation of nuclear functions. Biochem. J.

[19] McDaid JR, Loughery J, Dunne P (2009). MLH1 mediates PARP-dependent cell death in response to the methylating agent N-methyl-N-nitrosourea. Br. J. Cancer.

[20] Malanga M, Althaus FR (2005). The role of poly(ADP-ribose) in the DNA damage signaling network. Biochem. Cell Biol.

[21] Rouleau M, Aubin RA, Poirier GG (2004). Poly(ADP-ribosyl)ated chromatin domains: access granted. J. Cell Sci.

[22] Shim KS, Bergelson JM, Furuse M, Ovod V, Krude T, Lubec G (2003). Reduction of chromatin assembly factor 1 p60 and C21orf2 protein, encoded on chromosome 21, in Down syndrome brain. J. Neural Transm. Suppl.

[23] Das C, Lucia MS, Hansen KC, Tyler JK (2009). CBP/p300-mediated acetylation of histone H3 on lysine 56. Nature.

[24] Li Q, Huang Y, Xiao N, Murray V, Chen J, Wang J (2008). Real time investigation of protein folding, structure, and dynamics in living cells. Methods Cell Biol.

[25] Chen CC, Tyler J (2008). Chromatin reassembly signals the end of DNA repair. Cell Cycle.

[26] Kadyrova LY, Blanko ER, Kadyrov FA (2011). CAF-I-dependent control of degradation of the discontinuous strands during mismatch repair. Proc. Natl. Acad. Sci. USA.

[27] Kim JA, Haber JE (2009). Chromatin assembly factors Asf1 and CAF-1 have overlapping roles in deactivating the DNA damage checkpoint when DNA repair is complete. Proc. Natl. Acad. Sci. USA.

[28] Takami Y, Ono T, Fukagawa T, Shibahara K, Nakayama T (2007). Essential role of chromatin assembly factor-1-mediated rapid nucleosome assembly for DNA replication and cell division in vertebrate cells. Mol. Biol. Cell.

[29] Nabatiyan A, Krude T (2004). Silencing of chromatin assembly factor 1 in human cells leads to cell death and loss of chromatin assembly during DNA synthesis. Mol. Cell. Biol.

[30] Hoek M, Stillman B (2003). Chromatin assembly factor 1 is essential and couples chromatin assembly to DNA replication in vivo. Proc. Natl. Acad. Sci. USA.

[31] Polo SE, Theocharis SE, Grandin L (2010). Clinical significance and prognostic value of chromatin assembly factor-1 overexpression in human solid tumours. Histopathology.

[32] Staibano S, Mascolo M, Rocco A (2011). The proliferation marker chromatin assembly factor-1 is of clinical value in predicting the biological behaviour of salivary gland tumours. Oncol. Rep.

[33] Staibano S, Mascolo M, Mancini FP (2009). Overexpression of chromatin assembly factor-1 (CAF-1) p60 is predictive of adverse behaviour of prostatic cancer. Histopathology.

[34] Staibano S, Mignogna C, Lo Muzio L (2007). Chromatin assembly factor-1 (CAF-1)-mediated regulation of cell proliferation and DNA repair: a link with the biological behaviour of squamous cell carcinoma of the tongue?. Histopathology.

[35] Cheng JX, Liu BL, Zhang X (2009). How powerful is CD133 as a cancer stem cell marker in brain tumors?. Cancer Treat. Rev.

[36] Gillison ML (2009). HPV and prognosis for patients with oropharynx cancer. Eur. J. Cancer.

[37] Gillison M (2010). HPV and its effect on head and neck cancer prognosis. Clin. Adv. Hematol. Oncol.

[38] Gillison ML, D’Souza G, Westra W (2008). Distinct risk factor profiles for human papillomavirus type 16-positive and human papillomavirus type 16-negative head and neck cancers. J. Natl Cancer Inst.

[39] Kumar B, Cordell KG, Lee JS (2008). EGFR, p16, HPV Titer, Bcl-xL and p53, sex, and smoking as indicators of response to therapy and survival in oropharyngeal cancer. J. Clin. Oncol.

[40] Jung AC, Briolat J, Millon R (2010). Biological and clinical relevance of transcriptionally active human papillomavirus (HPV) infection in oropharynx squamous cell carcinoma. Int. J. Cancer.

[41] Snow A, Laudadio J (2010). Human papillomavirus detection in head and neck squamous cell carcinomas. Adv. Anat. Pathol.

[42] Hoffmann M, Ihloff AS, Görögh T (2010). p16(INK4a) overexpression predicts translational active human papillomavirus infection in tonsillar cancer. Int. J. Cancer.

[43] Singhi AD, Westra WH (2010). Comparison of human papillomavirus in situ hybridization and p16 immunohistochemistry in the detection of human papillomavirus-associated head and neck cancer based on a prospective clinical experience. Cancer.

[44] American Joint Committee on Cancer (2009). AJCC cancer staging manual.

[45] Wang SL, Yang CH, Chen HH, Chai CY (2006). A simple and economical method for the manual construction of well-aligned tissue arrays. Pathol. Res. Pract.

[46] Oliveira-Costa JP, Zanetti JS, Silveira GG (2011). Differential expression of HIF-1α in CD44+CD24–/low breast ductal carcinomas. Diagn. Pathol.

[47] Polo SE, Theocharis SE, Klijanienko J (2004). Chromatin assembly factor-1, a marker of clinical value to distinguish quiescent from proliferating cells. Cancer Res.

[48] Staibano S, Pepe S, Lo Muzio L (2005). Poly(adenosine diphosphate-ribose) polymerase 1 expression in malignant melanomas from photoexposed areas of the head and neck region. Hum. Pathol.

[49] Volgareva G, Zavalishina L, Andreeva Y (2004). Protein p16 as a marker of dysplastic and neoplastic alterations in cervical epithelial cells. BMC Cancer.

[50] Ordi J, Garcia S, del Pino M (2009). p16 INK4a immunostaining identifies occult CIN lesions in HPV-positive women. Int. J. Gynecol. Pathol.

[51] Sabol I, Salakova M, Smahelova J (2008). Evaluation of different techniques for identification of human papillomavirus types of low prevalence. J. Clin. Microbiol.

[52] Tan SE, Garland SM, Rumbold AR, Tabrizi SN (2010). Human papillomavirus genotyping using archival vulval dysplastic or neoplastic biopsy tissues: comparison between the INNO-LiPA and linear array assays. J. Clin. Microbiol.

[53] Celetti A, Testa D, Staibano S (2005). Overexpression of the cytokine osteopontin identifies aggressive laryngeal squamous cell carcinomas and enhances carcinoma cell proliferation and invasiveness. Clin. Cancer Res.

[54] Romano S, D’Angelillo A, Pacelli R (2010). Role of FK506 binding protein 51 (FKBP51) in the control of apoptosis of irradiated melanoma cells. Cell Death Differ.

[55] Landis JR, Koch GG (1997). The measurement of observer agreement for categorical data. Biometrics.

[56] Costea DE, Tsinkalovsky O, Vintermyr OK, Johannessen AC, Mackenzie IC (2006). Cancer stem cells—new and potentially important targets for the therapy of OSCC. Oral Dis.

[57] Maitland NJ, Collins A (2005). A tumour stem cell hypothesis for the origins of prostate cancer. BJU Int.

[58] Hirschmann-Jax C, Foster AE, Wulf GG (2004). A distinct ‘side population’ of cells with high drug efflux capacity in human tumor cells. Proc. Natl. Acad. Sci. USA.

[59] Pollard SM, Yoshikawa K, Clarke ID (2009). Glioma stem cell lines expanded in adherent culture have tumor-specific phenotypes and are suitable for chemical and genetic screens. Cell Stem Cell.

[60] Hamburger AW, Salmon SE (1977). Primary bioassay of human tumor stem cells. Science.

[61] Reya T, Morrison SJ, Clarke MF, Weissman IL (2001). Stem cells, cancer, and cancer stem cells. Nature.

[62] Tabor MP, Brakenhoff RH, van Houten VM (2001). Persistence of genetically altered fields in head and neck cancer patients: biological and clinical implications. Clin. Cancer Res.

[63] Gupta PB, Onder TT, Jiang G (2009). Identification of selective inhibitors of cancer stem cells by high-throughput screening. Cell.

[64] Bomken S, Fiser K, Heidenreich O, Vormoor J (2010). Understanding the cancer stem cell. Br. J. Cancer.

[65] Fukushima M, Kuzuya K, Ota K, Ikai K (1981). Poly(ADP-ribose) synthesis in human cervical cancer cell—diagnostic cytological usefulness. Cancer Lett.

[66] Alderson T (1990). New targets for cancer chemotherapy—poly(ADP-ribosylation) processing and polyisoprene metabolism. Biol. Rev. Camb. Philos. Soc.

[67] Berger NA, Adams JW, Sikorski GW, Petzold SJ, Shearer WT (1978). Synthesis of DNA and poly(adenosine diphosphate ribose) in normal and chronic lymphocytic leukemia lymphocytes. J. Clin. Invest.

[68] Hirai K, Ueda K, Hayaishi O (1983). Aberration of poly(adenosine diphosphate-ribose) metabolism in human colon adenomatous polyps and cancers. Cancer Res.

[69] Khan K, Araki K, Wang D (2010). Head and neck cancer radiosensitization by the novel poly(ADP-ribose) polymerase inhibitor GPI-15427. Head Neck.

[70] Helmbach H, Kern MA, Rossmann E (2002). Drug resistance towards etoposide and cisplatin in human melanoma cells is associated with drug-dependent apoptosis deficiency. J. Invest. Dermatol.

[71] Virág L (2005). Structure and function of poly(ADP-ribose) polymerase-1: role in oxidative stress-related pathologies. Curr. Vasc. Pharmacol.

[72] Tang L, Tron VA, Reed JC (1998). Expression of apoptosis regulators in cutaneous malignant melanoma. Clin. Cancer Res.

[73] Decker P, Muller S (2002). Modulating poly (ADP-ribose) polymerase activity: potential for the prevention and therapy of pathogenic situations involving DNA damage and oxidative stress. Curr. Pharm. Biotechnol.

[74] Bürkle A, Brabeck C, Diefenbach J, Beneke S (2005). The emerging role of poly(ADP-ribose) polymerase-1 in longevity. Int. J. Biochem. Cell Biol.

[75] Rojo F, García-Parra J, Zazo S (2011). Nuclear PARP-protein overexpression is associated with poor overall survival in early breast cancer. Ann. Oncol.

[76] Brustmann H (2007). Poly(adenosine diphosphate-ribose) polymerase expression in serous ovarian carcinoma: correlation with p53, MIB-1, and outcome. Int. J. Gynecol. Pathol.

[77] Bouda M, Gorgoulis VG, Kastrinakis NG (2000). ‘High risk’ HPV types are frequently detected in potentially malignant and malignant oral lesions, but not in normal oral mucosa. Mod. Pathol.

[78] Sugiyama M, Bhawal UK, Dohmen T, Ono S, Miyauchi M, Ishikawa T (2003). Detection of human papillomavirus-16 and HPV-18 DNA in normal, dysplastic, and malignant oral epithelium. Oral Surg. Oral Med. Oral Pathol. Oral Radiol. Endod.

[79] D’Souza G, Kreimer AR, Viscidi R (2007). Case-control study of human papillomavirus and oropharyngeal cancer. N. Engl. J. Med.

[80] Luo CW, Roan CH, Liu CJ (2007). Human papillomaviruses in oral squamous cell carcinoma and pre-cancerous lesions detected by PCR-based gene-chip array. Int. J. Oral Maxillofac. Surg.

[81] Chow LT, Broker TR, Steinberg BM (2010). The natural history of human papillomavirus infections of the mucosal epithelia. APMIS.

[82] Allam JP, Erdsach T, Wenghoefer M, Bieber T, Appel TR, Novak N (2008). Successful treatment of extensive human papillomavirus-associated oral leucoplakia with imiquimod. Br. J. Dermatol.

[83] Varnai AD, Bollmann M, Bankfalvi A (2009). The prevalence and distribution of human papillomavirus genotypes in oral epithelial hyperplasia: proposal of a concept. J. Oral Pathol. Med.

[84] Worden FP, Kumar B, Lee JS (2008). Chemoselection as a strategy for organ preservation in advanced oropharynx cancer: response and survival positively associated with HPV16 copy number. J. Clin. Oncol.

[85] Boyer SN, Wazer DE, Band V (1996). E7 protein of human papilloma virus-16 induces degradation of retinoblastoma protein through the ubiquitin–proteasome pathway. Cancer Res.

[86] Iwasaki I, Suda T (2009). Cancer stem cells and their niche. Cancer Sci.

[87] Heck JE, Barthiller J, Vaccarella S (2010). Sexual behaviours and the risk of head and neck cancers: a pooled analysis in the International Head and Neck Cancer Epidemiology (INHANCE) consortium. Int. J. Epidemiol.

[88] Lassen P, Eriksen JG, Hamilton-Dutoit S, Tramm T, Alsner J, Overgaard J (2009). Effect of HPV-associated p16INK4A expression on response to radiotherapy and survival in squamous cell carcinoma of the head and neck. J. Clin. Oncol.

[89] Peralta-Leal A, Rodríguez-Vargas JM, Aguilar-Quesada R (2009). PARP inhibitors: new partners in the therapy of cancer and inflammatory diseases. Free Radic. Biol. Med.

[90] Soldatenkov VA, Smulson M (2000). Poly(ADP-ribose) polymerase in DNA damage-response pathway: implications for radiation oncology. Int. J. Cancer.

[91] Szabo C, Dawson VL (1998). Role of poly(ADP-ribose) synthetase in inflammation and ischaemia–reperfusion. Trends Pharmacol. Sci.

[92] Saini V, Shoemaker RH (2010). Potential for therapeutic targeting of tumor stem cells. Cancer Sci.

[93] Sharma SV, Lee DY, Li B (2010). A chromatin-mediated reversible drug-tolerant state in cancer cell subpopulations. Cell.

[94] Kirkegaard T, Edwards J, Tovey S (2006). Observer variation in immunohistochemical analysis of protein expression, time for a change?. Histopathology.

