# Supplementary material for: Overexpression of chromatin assembly factor-1 p60, poly(ADP-ribose) polymerase 1 and nestin predicts metastasizing behaviour of oral cancer
Source: Histopathology. 2012 Dec;61(6):1089–105. doi: 10.1111/j.1365-2559.2012.04313.x (PMC3546388; doi:10.1111/j.1365-2559.2012.04313.x)
Supplement: Supplementary file 1 [file his0061-1089-SD1.doc]

**Supporting information.**

**Supplementary material and methods**

No patient experienced radiotherapy before surgery.

The study design and procedures involving tissue samples collection and handling were performed according to the Declaration of Helsinki, in agreement with the current Italian law, and to the Institutional Ethical Committee guidelines.

*Tissue microarray (TMA) construction*

The most representative tumour area for each case was selected on a donor tissue block under the guidance of the corresponding haematoxylin and eosin (H&E) section. A manual tissue microarrayer (Tissue-Tek Quick-Ray Sakura, Torrance, Ca, USA) was used to punch one cylindrical core tissue specimen (3 mm diameter) from the selected area. The cores were implanted into a recipient paraffin block to construct the TMA, each containing 2 samples of non-neoplastic oral mucosa (control). TMAs were chilled at -10°C for 30 min.^43^ Then, several 4-µm sections from each TMA were cut. The first section was stained with H&E to confirm both the presence of the selected areas from each tumour and the integrity of tissues. The other sections were mounted on poly-lysine coated glass slides.

*Immunohistochemistry*

The slides were heated at 55°C for 60 min, deparaffinized, and processed for antigen retrieval by microwave oven (5 min/3 times, in 1% sodium citrate buffer, pH 6.0). Non-specific bindings were blocked with 1.5 % non-immune mouse serum (1:20, Dakopatts, Hamburg, Germany, 30 min at room temperature). Endogenous activities (peroxidase and alkaline phosphatase) were quenched with dual endogenous enzyme block (0.5% H^2^O^2^ in methanol and detergent); after two rinses with T-TBS buffer, sections were incubated with the all primary antibodies (Table 2). The binding of primary antibodies was amplified with the appropriate secondary antibody and detected with diaminobenzidine and permanent red. After nuclear counterstaining with Mayer's hematoxylin for 30 sec, sections were mounted and cover-slipped with a synthetic medium.

For negative controls, non immune serum in T-TBS buffer (1:500) was used instead of the primary antibodies.

*HPV genotyping*

DNA isolation and HPV genotyping were performed by using the INNO-LiPA PCR-based HPV Genotyping Extra test (Innogenetics Biotechnology for Healthcare, Gent, Belgium).^51, 52^ This test identifies 28 HPV genotypes: 6, 11, 16, 18, 26, 31, 33, 35, 39, 40, 43, 44, 45, 52, 53, 54, 56, 58, 59, 66, 68, 69, 70, 71, 73, 74, and 82.

*Cell lines*

Normal human keratinocytes (HNEK) were cultured in keratinocyte growth medium (Cambrex, East Rutherford, NJ, USA). BHY, CAL33 and HN cell lines were described elsewhere, according to manufacturer’s recommendation.^53^ HN and BHY cell lines were derived from a human oral cavity SCC, CAL33 cells from human tongue SCC. Cells were maintained in DMEM supplemented with 10% fetal bovine serum, 2 mM L-glutamine and 100 units/ml penicillin-streptomycin (GIBCO, Paisley, PA, USA). The HaCat cells were derived from in vitro spontaneously transformed keratinocytes from histologically normal skin.

*Protein studies*

Immunoblotting experiments were performed according to standard procedures.^54^ For tissue protein extraction, samples were snap-frozen and immediately homogenized in lysis buffer by using the Mixer Mill apparatus (Qiagen). Protein quantified by a modified Bradford assay (Bio-Rad). Antigens were revealed by an enhanced chemiluminescence detection kit (ECL, Amersham).

*Analysis of cell death*

HaCaT (immortalized keratinocytes) and Cal 33 (squamous cell carcinoma of tongue) were transfected with specific short interfering oligoribonucleotide (siRNA), corresponding to human cDNA sequences for Caf p60 (Qiagen Germantown, Philadelphia, PA, USA) or with a non-silencing RNA (AllStars neg control siRNA, Qiagen) as control. For transfection details see[62]. After 24h, cells were treated with the PARP-1 inhibitor PJ34 (1) (Alexis,Vinci-Biochem, Firenze, Italy) at the doses of 0.5, 5 and 50 microM. Analysis of DNA content was performed by propidium iodide incorporation as described [62].

**Reference**

*(Note: reference number corresponds to reference list in main article)*

45.Wang, SL, Yang CH, Chen HH, *et al*. A simple and economical method for the manual construction of well-aligned tissue arrays. *Pathol Res Pract* 2006; **202**:485-486.

51.Sabol I, Salakova M, Smahelova J, *et al*. Evaluation of different techniques for identification of humanpapillomavirus types of low prevalence. *J Clin Microbiol* 2008; **46**:1606-1613.

52.Tan SE, Garland SM, Rumbold AR, *et al*. Human papillomavirus genotyping using archival vulval dysplastic or neoplastic biopsy tissues: comparison between the INNO-LiPA and linear array assays. *J Clin Microbiol* 2010; **48**:1458-1460.

53.Celetti A, Testa D, Staibano S, *et al*. Overexpression of the cytokine osteopontin identifies aggressive laryngeal squamous cell carcinomas and enhances carcinoma cell proliferation and invasiveness. *Clin Cancer Res* 2005; **11**:8019-8027.

54.Romano S, D'Angelillo A, Pacelli R, *et al.* Role of FK506 binding protein 51 (FKBP51) in the Control of Apoptosis of Irradiated Melanoma Cells. *Cell Death and Differentiation* 2010; **17:** 145-157.

**Supplementary tables**

**Table S1**: Correlation between sex, age, localization, grading and staging and an adverse event (recurrence, metastasis, death for disease)

|  | | **No Event** | **Any Event** | **Difference** |
| --- | --- | --- | --- | --- |
| **Males (%)** | | 31 (47.0%) | 10 (15.1%) | *NS* |
| **Mean Age (SE)** | |  |  |  |
| **Localization** | **0** | 2 (3.8%) | 2 (14.3%) | *NS* |
|  | **1** | 33 (63.5%) | 8 (57.1%) |  |
|  | **2** | 12 (23.1%) | 4 (28.6%) |  |
|  | **3** | 5 (9.6%) | 0 (0%) |  |
| **Grade** | **0** | 7 (13.5%) | 1 (7.1%) | *NS* |
|  | **1** | 19 (36.6%) | 6 (42.9%) |  |
|  | **2** | 26 (50.0%) | 7 (50.0%) |  |
| **Stage** | **0** | 18 (34.6%) | 3 (21.4%) | *NS* |
|  | **1** | 12 (23.1%) | 7 (50.0%) |  |
|  | **2** | 4 (7.7%) | 0 (0%) |  |
|  | **3** | 18 (34.6%) | 4 (28.6%) |  |

NS: not significant

Subsite: 0: palate; 1: tong; 2: floor; 3: lip

**Table S2:** Correlation between all markers expression and relapse

|  | | **No Relapse** | **Relapse** | **Difference** |
| --- | --- | --- | --- | --- |
| **CAF-1/p60** | **LOW** | 33 (56%) | 0 (0%) | *p<0.001* |
|  | **HIGH** | 26 (44%) | 7 (100%) |  |
| **PARP-1** | **LOW** | 28 (47%) | 0 (0%) | *p<0.001* |
|  | **HIGH** | 31 (53%) | 7 (100%) |  |
| **P16** | **0** | 49 (83%) | 7 (100%) | *NS* |
|  | **1** | 10 (17%) | 0 (0%) |  |
| **HPV** | **0** | 49 (83%) | 7 (100%) | *NS* |
|  | **1** | 10 (17%) | 0 (0%) |  |
| **CD166** | **LOW** | 34 (58%) | 0 (0%) | *p<0.001* |
|  | **HIGH** | 25 (42%) | 7 (100%) |  |
| **CD133** | **LOW** | 31 (53%) | 2 (29%) | *NS* |
|  | **HIGH** | 28 (47%) | 5 (71%) |  |
| **Nestin** | **LOW** | 29 (49%) | 0 (0%) | *p<0.001* |
|  | **HIGH** | 30 (51%) | 7 (100%) |  |
| **CD44** | **LOW** | 28 (47%) | 2 (29%) | *NS* |
|  | **HIGH** | 31 (53%) | 5 (71%) |  |
| **CD44v6** | **LOW** | 13 (22%) | 2 (29%) | *NS* |
|  | **HIGH** | 46 (78%) | 5 (71%) |  |

NS: not significant

**Table S3:** Correlation between all markers expression and metastasis

|  | | **No Metastasis** | **Metastasis** | **Difference** |
| --- | --- | --- | --- | --- |
| **CAF-1/p60** | **LOW** | 33 (56%) | 0 (0%) | *p<0.001* |
|  | **HIGH** | 26 (44%) | 7 (100%) |  |
| **PARP-1** | **LOW** | 28 (47%) | 0 (0%) | *p<0.001* |
|  | **HIGH** | 31 (53%) | 7 (100%) |  |
| **P16** | **0** | 49 (83%) | 7 (100%) | *NS* |
|  | **1** | 10 (17%) | 0 (0%) |  |
| **HPV** | **0** | 49 (83%) | 7 (100%) | *NS.* |
|  | **1** | 10 (17%) | 0 (0%) |  |
| **CD166** | **LOW** | 34 (58%) | 0 (0%) | *p<0.001* |
|  | **HIGH** | 25 (42%) | 7 (100%) |  |
| **CD133** | **LOW** | 31 (53%) | 2 (29%) | *NS* |
|  | **HIGH** | 28 (47%) | 5 (71%) |  |
| **Nestin** | **LOW** | 29 (49%) | 0 (0%) | *p<0.001* |
|  | **HIGH** | 30 (51%) | 7 (100%) |  |
| **CD44** | **LOW** | 30 (51%) | 0 (0%) | *p<0.001* |
|  | **HIGH** | 29 (49%) | 7 (100%) |  |
| **CD44v6** | **LOW** | 15 (25%) | 0 (0%) | *p<0.001* |
|  | **HIGH** | 44 (75%) | 7 (100%) |  |

NS: not significant

**Table S4:** Correlation between all markers expression and death for disease

|  | | **No Death** | **Death** | **Difference** |
| --- | --- | --- | --- | --- |
| **CAF-1/p60** | **LOW** | 33 (59%) | 0 (0%) | *p<0.001* |
|  | **HIGH** | 23 (41%) | 10 (100%) |  |
| **PARP-1** | **LOW** | 28 (50%) | 0 (0%) | *p<0.001* |
|  | **HIGH** | 28 (50%) | 10 (100%) |  |
| **P16** | **0** | 46 (82%) | 10 (100%) | *NS* |
|  | **1** | 10 (18%) | 0 (0%) |  |
| **HPV** | **0** | 46 (82%) | 10 (100%) | *NS* |
|  | **1** | 10 (18%) | 0 (0%) |  |
| **CD166** | **LOW** | 33 (59%) | 1 (10%) | *p<0.001* |
|  | **HIGH** | 23 (41%) | 9 (90%) |  |
| **CD133** | **LOW** | 31 (55%) | 2 (20%) | *p=0.02* |
|  | **HIGH** | 25 (45%) | 8 (80%) |  |
| **Nestin** | **LOW** | 29 (52%) | 0 (0%) | *p<0.001* |
|  | **HIGH** | 27 (48%) | 10 (100%) |  |
| **CD44** | **LOW** | 30 (54%) | 0 (0%) | *p<0.001* |
|  | **HIGH** | 26 (46%) | 10 (100%) |  |
| **CD44v6** | **LOW** | 15 (27%) | 0 (0%) | *p<0.001* |
|  | **HIGH** | 41 (73%) | 10 (100%) |  |

NS: not significant
